# Navigating the COVID-19 Therapeutic Landscape: Unveiling Novel Perspectives on FDA-Approved Medications, Vaccination Targets, and Emerging Novel Strategies

**DOI:** 10.3390/molecules29235564

**Published:** 2024-11-25

**Authors:** Reham F. Barghash, Donato Gemmati, Ahmed M. Awad, Mustafa M. M. Elbakry, Veronica Tisato, Kareem Awad, Ajay Vikram Singh

**Affiliations:** 1 Institute of Chemical Industries Research, National Research Centre, Dokki, Cairo 12622, Egypt; 2 Faculty of Biotechnology, October University for Modern Sciences and Arts (MSA), Cairo 12451, Egypt; 3 Department of Translational Medicine, University of Ferrara, 44121 Ferrara, Italy; 4 Department of Chemistry, California State University Channel Islands, Camarillo, CA 93012, USA; 5 Biochemistry Department, Faculty of Science, Ain Shams University, Cairo 11566, Egypt; 6 Centre Hemostasis & Thrombosis, University of Ferrara, 44121 Ferrara, Italy; 7 Institute of Pharmaceutical and Drug Industries Research, National Research Center, Dokki, Cairo 12622, Egypt; kareem.awad@web.de; 8 Department of Chemical and Product Safety, German Federal Institute for Risk Assessment (BfR), Max-Dohrn-Strasse 8-10, 10589 Berlin, Germany

**Keywords:** molnupiravir, SARS-CoV-2, drug repurposing, vaccines, variant of concerns (VOC), paxlovid

## Abstract

Amidst the ongoing global challenge of the SARS-CoV-2 pandemic, the quest for effective antiviral medications remains paramount. This comprehensive review delves into the dynamic landscape of FDA-approved medications repurposed for COVID-19, categorized as antiviral and non-antiviral agents. Our focus extends beyond conventional narratives, encompassing vaccination targets, repurposing efficacy, clinical studies, innovative treatment modalities, and future outlooks. Unveiling the genomic intricacies of SARS-CoV-2 variants, including the WHO-designated Omicron variant, we explore diverse antiviral categories such as fusion inhibitors, protease inhibitors, transcription inhibitors, neuraminidase inhibitors, nucleoside reverse transcriptase, and non-antiviral interventions like importin α/β1-mediated nuclear import inhibitors, neutralizing antibodies, and convalescent plasma. Notably, Molnupiravir emerges as a pivotal player, now licensed in the UK. This review offers a fresh perspective on the historical evolution of COVID-19 therapeutics, from repurposing endeavors to the latest developments in oral anti-SARS-CoV-2 treatments, ushering in a new era of hope in the battle against the pandemic.

## 1. Introduction

Coronaviruses (CoVs) are enveloped RNA viruses and are members of the order Nidovirales’ Coronaviridae family that cause respiratory, hepatic, neurological, and intestinal disease [1]. Four endemic coronaviruses in humans, known as CoV-229E, CoV-OC43, CoV-NL63, and CoV-HKU1, are frequently linked to mild respiratory illness in healthy people [2]. Coronaviruses, such as the Middle East Respiratory Syndrome (MERS-CoVs), Severe Acute Respiratory Syndrome (SARS-CoV) [3], and, eventually, the new Severe Acute Respiratory Syndrome (SARS-CoV-2) that cause COVID-19, are all examples of deadly outbreaks caused by coronaviruses [4,5,6,7,8]. COVID-19 was identified in Wuhan, China, by December 2019 [3,4,9,10,11,12]. As a result, the World Health Organization (WHO) directed various tests about the recent onset of this outbreak [12,13]. The COVID-19 outbreak initially emerged from an unidentified animal source at a market [12], with evidence suggesting zoonotic transmission [14], likely involving intermediate hosts such as bats [10]. SARS-CoV-2 is a positive, single-RNA-stranded virus that has the potential to infect humans or animals. SARS-CoV-2 belongs to the Beta-CoV subfamily, one of the four CoV subfamilies: Gamma, Delta, Alpha, and Beta. The Beta and Alpha CoV viruses attack mammals, but the Delta and Gamma CoV viruses only affect birds [8,15,16]. SARS-CoV-2 exhibits persistent transmission from person to person via direct/indirect contact and through the environment as respiratory droplets and/or aerosols [17,18,19]. Research on SARS-CoV-2 suggests that most cases are linked to relatively low viral loads, resulting in a range of symptoms with varying durations [20]. The onset of more severe virus symptoms with a larger load can be seen in common symptoms such as fever and cough [3,14]. Some symptoms, including fever, inflammatory reactions, pneumonia, and hypoxemia, may manifest as the illness worsens. Most COVID-19 patients either show no symptoms or have mild illness, and respiratory patients should therefore visit hospitals [5,20,21,22]. Since December 2019, COVID-19 has been a significant public concern around the globe. Since December 2019, COVID-19 has been a significant public concern worldwide. By November 9, 2021, over 250 million COVID-19 cases had been reported across 224 countries and territories [23]. The discovery of this novel virus prompted researchers to develop and test new vaccines to ensure their effectiveness [24]. It is crucial to draw attention to the SARS-CoV-2 variations discovered in the genomes of SARS-CoV-2 virions. These variations are anticipated to have an advantageous effect on the phenotype of the virus in some circumstances. Such modifications may have an impact on the pathogenicity, transmissibility, infectiousness, as well as antigenicity of viruses [25]. On 24 November 2021, the World Health Organization classified the SARS-CoV-2 Omicron variant B.1.1.529, which appeared in South Africa, as a variation under monitoring (VUM). Two days later, the Omicron version was classified as a variant of concern (VOC). This variant has several mutations, with about 15 changes to the spike receptor-binding domain (RBD) [26].

There have been various proposed techniques to combat SARS-CoV-2. Among these, targeting ACE2 either directly through supplements or inadvertently through medications [27]. Additionally, the main protease (Mpro) has also been targeted to combat SARS-CoV-2 [7,28]. Based on the preceding, it is crucial to gather and comprehend the earlier articles written about the attempts and trials made to resist COVID-19 [29,30]. This article evaluates the majority of previously repurposed FDA-approved medications, their clinical studies, and the most recent possibilities for fighting SARS-CoV-2. This article could help researchers from around the globe in developing a comprehensive understanding of this pandemic and potential therapeutic approaches.

## 2. Vaccines Targets for SARS-CoV-2

**Figure 1:**
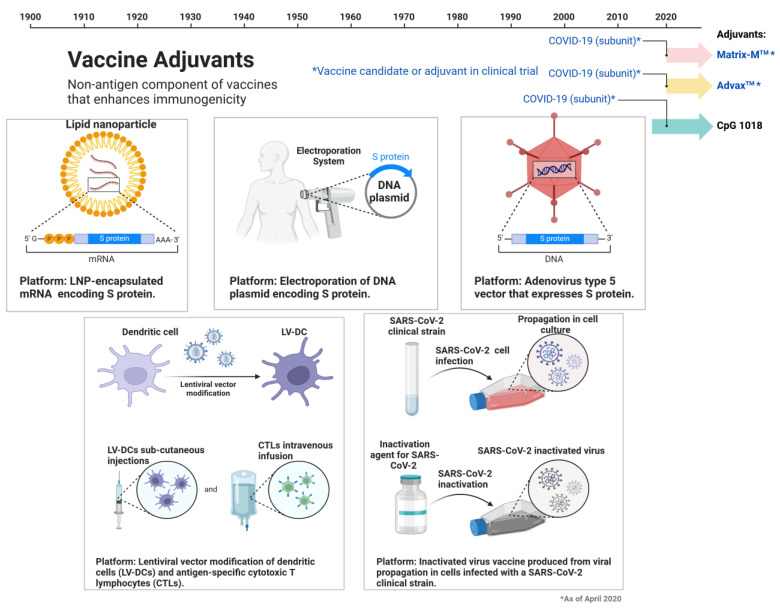
Schematic diagram for vaccine candidates in human trial. Created with Biorender. The timeline could reflect key milestones in the development, approval, and rollout of COVID-19 vaccines across different platforms, highlighting significant events. January 2020: Identification of SARS-CoV-2 and global initiation of vaccine research. March 2020: Start of clinical trials for multiple vaccine platforms (e.g., mRNA, adenovirus vector, protein subunit). December 2020: Emergency Use Authorization (EUA) of Pfizer-BioNTech (Comirnaty) and Moderna (Spikevax) mRNA vaccines in the United States and Europe. February 2021: EUA for Johnson & Johnson’s adenovirus vector vaccine. March 2021: WHO Emergency Use Listing (EUL) for AstraZeneca (Vaxzevria) adenovirus vector vaccine. July 2021: Full FDA approval of Pfizer-BioNTech vaccine (Comirnaty) for individuals aged 16+. November 2021: Booster doses recommended due to waning immunity against new variants, including Delta. January 2022: Development and testing of variant-specific vaccine updates, especially targeting Omicron.

Like other viral diseases, vaccination is the leading way to avoid COVID-19. Several vaccine platforms have been created since the SARS-CoV-2 emergency, and as of July 2022, about 40 vaccines received global approval. Mainly, 196 vaccines are in preclinical trials, while 153 vaccines reached the clinical trials. The currently licensed vaccines are based on protein subunits (*n* = 16), inactivated virus (*n* = 11), nonreplicating viral vectors (*n* = 7), RNA (*n* = 4), DNA (*n* = 1), or virus-like particles (VLPs) (*n* = 1), Figure 1. The WHO issued Emergency Use Listing (EUL) for ten of these vaccinations, which are mentioned [31,32]. Vaccines utilizing protein subunits consist of antigenic fragments from pathogens, effectively preventing human viral infection [33]. However, they lack the full antigenic complexity of the virus, limiting their efficacy, as protection may be reduced due to a limited number of viral fragments [34]. Examples of protein subunit vaccines, such as COVOVAX (produced by the Serum Institute of India), the Novavax formulation, and Nuvaxovid (Novavax), involve the recombinant nanoparticle S protein linked to the Matrix-M adjuvant. The S protein has undergone stabilizing modifications designed to address the underlying issue of its conformational instability [35]. The inactivated vaccines, such as Covilo (Sinopharm, Shanghai, China), CoronaVac (Sinovac, Beijing, China), and Covaxin (Bharat Biotech, Turakapally, India) based on the whole virus in cells, followed by chemical inactivation, purification, and then mixing with particular substances that act as immune cell stimulants and immune response amplifiers, like aluminum hydroxide adjuvant [36]. It is known that pathogens that have been radioactively, chemically, or thermally inactivated occasionally lose their immunogenicity, making the platform less effective than those that use live attenuated pathogens [37]. Nonreplicated viral vector vaccines approved for human use rely on animal or human replication-defective adenovirus vectors. Notably, Vaxzevria (Oxford/AstraZeneca, Cambridge, UK) and Covishield, produced through the Oxford and AstraZeneca formulation by the Serum Institute of India and Fiocruz-Brazil, are licensed vaccines based on the chimpanzee adenovirus expressing the SARS-CoV-2 S glycoprotein. Additionally, Ad26.COV2.S, licensed by Janssen/Johnson & Johnson, New Brunswick, NJ, USA, utilizes a recombinant human adenovirus type 26 vector to express the S protein in a stable form [38]. Since RNA-based vaccines have been licensed for use in humans for the first time and have shown excellent safety and effectiveness profiles, this platform is leading the way in the rapid development of vaccinations against emerging cases [39,40,41]. Spikevax (Moderna, Cambridge, MA, USA) and Comirnaty (Pfizer/BioNTech, New York, NY, USA) are nucleoside-modified RNA vaccines formulated with lipid nanoparticles. They encode the full-length SARS-CoV-2 S protein, modified by two proline mutations to maintain the pre-fusion conformation. Despite variances in their engineering processes, both vaccines share this key feature. As per a recent meta-analysis report, out of the majority of vaccines, 81% had an effect against severe disease, still higher than 70% after getting complete vaccination associated with a 10% minimal reduction six months after immunization [42]. As most of these vaccines were developed using the prototype Wuhan-Hu-1 strain, they are less effective against the variants of concern (VOCs) that have surfaced since the pandemic’s inception. Therefore, to offer the best defense against these SARS-CoV-2 variations, modifications to vaccine composition to reflect the most common variant(s) of SARS-CoV-2 must be considered. Because COVID-19-vaccine-induced immunity is transient, new preventive measures that result in long-term protection are necessary.

Since the SARS-CoV-2 virus was identified in December 2019, viral genomes from global clinical samples have been sequenced, with thousands uploaded to public databases. Due to limited proofreading during genome replication, SARS-CoV-2, like other RNA viruses, exhibits a relatively high mutation rate [43]. However, the Coronaviridae family has a unique exoribonuclease moiety in the nsp14 protein [44], providing some proofreading capacity [45]. Initial studies indicated low nucleotide diversity, but diversity has risen with viral incidence.

**Figure 2:**
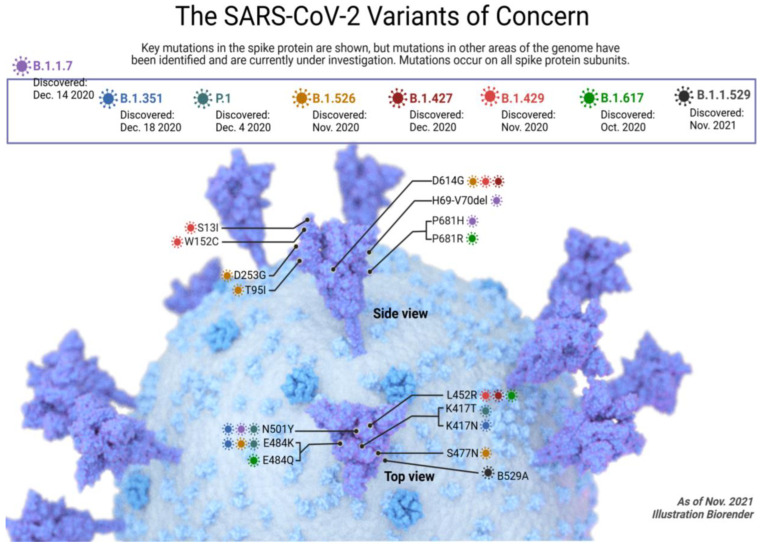
The SARS-CoV-2 variants of concern. Created with Biorender.

SARS-CoV-2 variants are classified by the CDC into three groups: variants of interest (VOIs), which may alter diagnostics or treatment sensitivity; variants of concern (VOCs), which show increased transmission, therapeutic failure, or reduced antibody neutralization; and variants of high consequence (VOHCs), for which medical countermeasures are less effective (none currently designated). Key mutations, such as D614G, first observed in early 2020, have contributed to enhanced replication and global spread. Five variants—Alpha, Beta, Gamma, Delta, and Omicron—are currently classified as VOCs [46,47,48] due to their transmissibility and impact on public health (Figure 2) [48,49].

The emergence and evolution of SARS-CoV-2 variants have significantly impacted the trajectory of the COVID-19 pandemic. The Alpha variant, initially identified in the UK as VUI-202012/01, designated B.1.1.7, and subsequently referred to as Alpha by the WHO, demonstrated increased transmissibility, higher viral loads, and a longer infectious period. Associated with elevated mortality, the Alpha variant posed challenges for detection due to S gene target failure (SGTF). Notably, it exhibited resistance to certain vaccines and therapeutic monoclonal antibodies. The Beta variant, B.1.351, originating in South Africa, showcased enhanced transmissibility and decreased neutralization by both convalescent sera and vaccines. The Gamma variant, P.1, identified in Brazil, presented heightened transmissibility, increased viral loads, and potential impacts on herd immunity. The Delta variant, B.1.617.2, contributed significantly to global transmission, displaying higher transmissibility and resistance to neutralization by certain antibodies and sera. Lastly, the Omicron variant, B.1.1, emerged in Botswana and South Africa, marking the fourth wave of the pandemic. Characterized by distinct biological traits, including strong ACE2 receptor binding, exceptional transmissibility, environmental stability, and resistance to authorized monoclonal antibodies, the Omicron variant poses new challenges to global efforts in combating COVID-19. The continuous monitoring of these variants, their interactions with existing treatments and vaccines, and the development of targeted interventions remain imperative for effective pandemic management.

JN.1, which first appeared in Denmark at the end of July, has quickly crossed international borders and been detected in a number of nations, including the United Kingdom, Canada, the United States, South Africa, Portugal, and Sweden. Numerous mutations inside the spike gene set this version apart and further complicate our knowledge of the virus’s activity. The WHO has made a noteworthy advancement in the fight against the virus by classifying the unique strain JN.1 as a “variant of interest”. This designation highlights the strain’s potential importance [50].

The five variants—Alpha, Beta, Gamma, Delta, and Omicron—which are currently classified as VOCs [46,47,48], are described as follows: 

### 2.1. Alpha SARS-CoV-2 Variant

In December 2020, a new SARS-CoV-2 variant, B.1.1.7 (Alpha), emerged in the UK and quickly became the predominant strain [51]. Characterized by 23 nucleotide mutations [52], including critical changes in the spike protein, Alpha demonstrated significantly higher transmissibility than earlier variants [53,54]. Studies estimate that its replication rate was 43% to 90% higher than prior strains, likely due to increased viral loads and longer infectious periods. These features contributed to elevated mortality rates and hospitalizations [55], particularly among patients aged 20–59.

The Alpha variant’s spike protein mutations also led to reduced detection in certain RT-qPCR tests, a phenomenon known as S gene target failure (SGTF) [56], which allowed for rapid identification in populations. Although Alpha showed partial resistance to some monoclonal antibody therapies [57,58], mRNA vaccines (Moderna’s mRNA-1273 and Pfizer-BioNTech’s BNT162b2) remained highly effective [59,60]. However, inactivated-virus vaccines like BBV152/COVAXIN (Bharat Biotech) and BBIBP-CorV (Sinopharm) [61,62] demonstrated reduced neutralizing efficacy. Other vaccines, such as AZD1222 (Oxford-AstraZeneca) and Sputnik V, exhibited moderate efficacy against the Alpha variant [63] but with some neutralization challenges, indicating that vaccine responses varied widely with this variant [64].

### 2.2. Beta SARS-CoV-2 Variant

Researchers from South Africa described another variation of SARS-CoV-2 that appeared following the initial epidemic wave in the same month of the initially detected Alpha variant in the UK [65], initially known as S501.V2, was referred as B.1.351 by Pango lineages and Beta by the WHO. When the Beta VOC was initially identified, it had 31 mutations, four of which were also present in the B.1 variant. There are 21 nonsynonymous mutations among the 27 unique variations reported in this lineage, while 12 have been fixed in the variant population over time [65]. The N501Y alteration on the S protein, critical for viral phenotype, is shared by this developing variation and the Alpha VOC. The Beta VOC was exhibited to be 50% more transmissible than previously circulating versions [66]. When compared to non-VOCs, Beta VOCs were associated with increased risk of hospitalization in European patients aged 40–59 and 60–79 years, as well as ICU with the 40–59 year age group; however, this did not increase deaths [55]. Beta VOCs’ decreased sensitivity to neutralization by recuperating and vaccine-elicited sera appear to be their most significant traits to date. The ability of the mRNA-1273, BNT162b2, BBIBP-CorV, CoronaVac, ChAdOx1, Sputnik V Ad26/Ad5, and nCoV-19/AZD1222 vaccines to neutralize this variation was less effective [58,62,64,67,68,69,70,71,72]. For example (ChAdOx1 nCoV-19/AZD1222 [72]), BNT162b2 appears to preserve its efficiency to prevent severe forms of the disease, despite a considerable decline in vaccination efficacy being seen in a population-based investigation [59]. According to assessment report EMA/158424/2021, the vaccines BBV152/COVAXIN and Ad26.COV2.S were evaluated to be effective against Beta VOC [73]. This variant decreased neutralizing by therapeutic monoclonal antibodies [67,71]. Therefore, the Beta VOC needs to be continuously monitored by genetic monitoring, as it may be linked to an increase in the frequency of reinfections and the failure of vaccines or treatments.

### 2.3. Gamma SARS-CoV-2 Variants

Another SARS-CoV-2 variation, known as P.1 (Gamma), was discovered in Manaus, Brazil in December 2020, which may have contributed to a significant rise in COVID-19 prevalence. Initially, this Gamma variant was identified by 35 mutations dispersed throughout the entire genome. The S gene contains 10 nonsynonymous mutations, of which 3 (K417T, E484 K, and N501Y) are shared with the variant B.1.351 and one (N501Y) is shared with both B.1.1.7 and B.1.351 variants [74]. The Gamma VOCs were the predominant variety in the city in January 2021 due to their estimated transmissibility, which was 1.7 to 2.5 times greater than those of the non-Gamma variants circulating in Manaus [74,75]. The increase in viral loads was also observed in Gamma variant-infected individuals, which may play a role in the more infectious behavior of this variant [75]. Gamma variant infection was linked to a significant probability of hospitalization and ICU admission [55]. The emergence of this variant may also be a factor in the reinfection of patients [76,77] and the recurrence of disease in regions where previous variants likely contributed to herd immunity [78]. When neutralized by convalescent plasma and therapeutic monoclonal antibodies, the Gamma variant is only partially to entirely susceptible. The vaccines mRNA-1273 and BNT162b2 fared the best, with slight to moderate declines in their ability to neutralize this variation [76,79,80,81]. A case of a patient who had the entire BNT162b2 vaccination and experienced modest symptoms following Gamma infection was documented [82]. CoronaVac’s effectiveness against Gamma was estimated, and AZD1222’s ability to destroy this virus was diminished [81].

### 2.4. Delta SARS-CoV-2 Variant

The Delta variant (B.1.617.2), first identified in India in late 2020, rapidly spread globally [83] and became the dominant SARS-CoV-2 lineage in many countries by mid-2021 [83,84]. Known for its high transmissibility, Delta had a reproduction number approximately 97% higher than that of non-VOCs [84] and significantly exceeded other variants of concern (VOCs) [85,86,87]. This increased transmissibility is largely attributed to key mutations in the spike protein, such as T478K and L452R, which enhance ACE2 binding and potentially improve viral entry into human cells. Delta’s rapid replication likely contributed to elevated viral loads, higher rates of hospitalization, and increased disease severity [85,88]. The Delta variant also demonstrated resistance to neutralization by several monoclonal antibodies and convalescent sera. While vaccines like Pfizer-BioNTech’s BNT162b2 and Moderna’s mRNA-1273 remained effective at preventing severe disease, partial immunization showed reduced neutralization capacity against Delta [89,90,91,92]. Fully vaccinated individuals, however, retained strong protection against severe illness. Inactivated-virus vaccines, including CoronaVac, Sinopharm, and BBV152/COVAXIN, showed varying levels of efficacy, with high effectiveness against severe disease [93] but lower performance in neutralizing Delta. Non-replicating viral vector vaccines, such as AZD1222, showed moderate to high efficacy, though effectiveness varied by population studies, warranting further investigation.

### 2.5. Omicron SARS-CoV-2 Variant

The emergence of the Omicron variant has significantly impacted the efficacy of existing COVID-19 therapies, particularly due to its increased transmissibility and multiple mutations in the spike protein. These mutations enhance Omicron’s binding affinity to the ACE2 receptor, facilitating rapid spread, even among vaccinated and previously infected individuals. Importantly, Omicron shows partial resistance to some therapeutic monoclonal antibodies that target the spike protein, diminishing their neutralization capacity. This partial resistance has necessitated updates in treatment protocols and sparked efforts to develop variant-specific antibodies and vaccines to better address Omicron and similar high-transmissibility variants [94,95].

## 3. Antiviral Drugs Against COVID-19

### 3.1. Fusion Inhibitors Targeting Spike Protein or **Viral Entry Inhibition**

**Figure 3:**
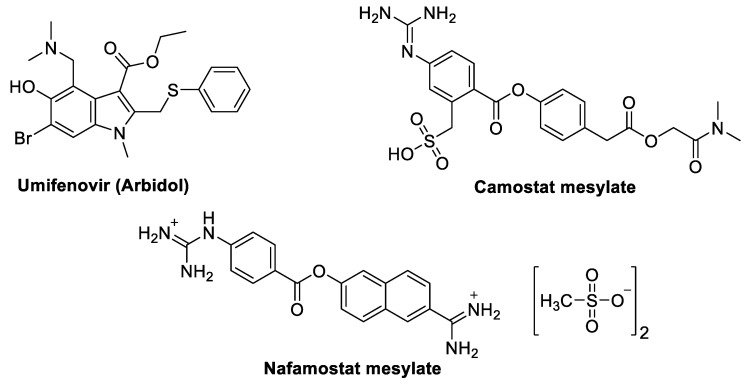
Chemical structures of fusion inhibitors that target S spike protein; Umifenovir, Camostat mesylate, and Nafamostat mesylate.

Viral entry inhibitors are a critical class of antiviral agents that prevent SARS-CoV-2 from penetrating host cells, effectively halting infection at its earliest stage. SARS-CoV-2 entry primarily depends on the binding of the viral spike (S) protein to the ACE2 receptor on human cells, followed by activation of this complex through the host cell protease TMPRSS2. This dual interaction not only facilitates viral fusion with the host cell membrane but also determines the efficiency of viral spread. Inhibitors targeting these pathways—such as Umifenovir, Camostat Mesylate, and Nafamostat (Figure 3)—have shown promise in blocking either spike protein binding or TMPRSS2 activity, thereby disrupting the viral life cycle before replication begins. The importance of viral entry inhibition has grown with the emergence of highly transmissible variants, like Delta and Omicron, which exhibit stronger ACE2 affinity and increased infectivity. By targeting early viral entry points, these inhibitors offer a potent means to reduce viral load and prevent the rapid spread of infection.

#### 3.1.1. Umifenovir (Arbidol)

A short indole derivative called umifenovir (Figure 3) has a variety of anti-RNA and anti-DNA viral effects. This inhibits viral internalization or attachment and blocks viral penetration into the host cell [96]. It is authorized to prevent and cure influenza A and B infections in China and Russia [97,98]. However, in vitro studies have shown activity against viral illnesses, including Hepatitis B and C viruses (HBV and HCV) and Ebola. Umifenovir was first authorized for influenza treatment in Russia in 1993. The drug is available in China and Russia for the treatment of upper respiratory influenza A and B infections [99]. Umifenovir is advised for COVID-19 patients in the “Treatment Scheme and New Coronavirus Pneumonia Diagnosis”. Adults receive 0.2 g of the drug three times per day for ten days [96]. In clinical trials, umifenovir, orally administrated, was found to reduce mortality and viral load in contrast to other unnamed antiviral medications or the interferon-only control group [100]. It includes mild general adverse effects, such as nausea, headaches, raised bilirubin, leukopenia, high alkaline phosphatase (AKP), and other symptoms like abdominal pain. No notable side effects were observed during treatment, according to retrospective studies to assess the safety and effectiveness of umifenovir therapy in COVID-19 patients [96].

#### 3.1.2. Camostat Mesylate

Camostat mesylate, another drug that targets virus fusion (Figure 3), is a serine protease inhibitor [101]. For target cell entry, SARS-CoV-2 binds to TMPRSS2 and/or ACE-2 receptors within the targeted host cells [102,103]. Camostat mesylate works by inhibiting TMPRSS2 [104]. The SARS-CoV-2 spike (S) protein is downregulated to prevent the virus from entering the cell and thereby preventing surface fusion [105]. An earlier study found that camostat mesylate prevented SARS-CoV from entering human epithelial bronchial cells [106]. In vitro studies revealed that camostat mesylate and E-64d, a cysteine protease inhibitor, effectively inhibit SARS-CoV-2 TMPRSS2 [107]. In Denmark and Germany, the effectiveness of hydroxychloroquine and camostat mesylate combination therapy has been evaluated [108]. Serine protease inhibitor, nafamostat mesylate, was found to have a 15-fold higher effectiveness for SARS-CoV-2 viral entrance into the host cells. Because nafamostat has more effective antiviral action and a good safety profile, it may be considered a safer alternative to camostat mesylate. Additionally, nafamostat mesylate is used to treat disseminated intravascular coagulation (DIC) with enhanced fibrinolysis seen in COVID-19 patients [108].

### 3.2. Protease Inhibitors

**Figure 4:**
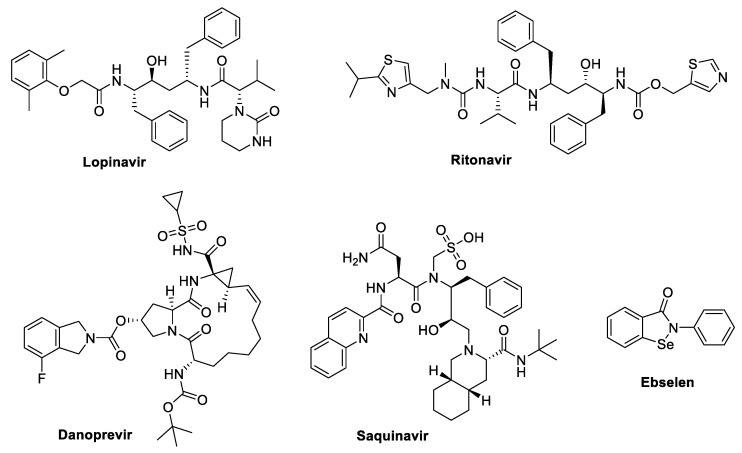
Chemical structures of protease inhibitors: Lopinavir, Ritonavir, Danoprevir, Saquinavir, and Ebselen.

The SARS-CoV-2 virus relies on two key proteases, the main protease (Mpro) and the papain-like protease (PLpro), for viral replication. These proteases play essential roles in processing the viral polyproteins and transforming them into functional units required for assembling new virus particles. Inhibitors targeting these proteases—such as Lopinavir, Ritonavir, Danoprevir, Saquinavir, and Ebselen, Figure 4—effectively disrupt the viral replication cycle, making them prime candidates for antiviral interventions.

#### 3.2.1. Lopinavir

In combination with lopinavir (Figure 4), Ritonavir is used for HIV infection treatment. Additionally, it has been shown that lopinavir inhibits SARS-CoV-2 replication with an EC50 ratio of 2.660/1.671 µM [109]. Half-maximal effective concentration (EC50). The number of eosinophils increased among COVID-19 cases after it was administered in China as an emergency medication [110]. Ritonavir and lopinavir, as HIV protease inhibitors, have shown encouraging results as SARS-CoV-2 major protease (Mpro) inhibitors in an in vitro investigation [11]. An earlier investigation revealed that the drug kaletra^®^, a specific lopinavir/ritonavir combination, has antiviral activity against the SARS-CoV in vitro and clinical studies [111]. As a result, this combination is also employed as a backup plan for COVID-19 patients. An earlier investigation showed that while lopinavir/ritonavir therapy was associated with better outcomes, it did not improve the patient’s clinical recovery from COVID-19 infection [112]. Although lopinavir’s efficacy for treating COVID-19 has not yet evaluated, but USA, Japan, and Singapore treat COVID-19 patients with such ritonavir/lopinavir combination. The lopinavir/ritonavir efficacy for COVID-19 is now being studied in clinical trials in many countries such as France, Spain, Thailand, China, Hong Kong, Canada, and the United States [108]. Furthermore, according to the WHO, a “solidarity” clinical trial for coronavirus was conducted using an inflammation-regulating molecule, interferon (INF)-β, alone or with lopinavir/ritonavir [109]. Additionally, patients with COVID-19 can experience improved clinical symptoms and lower viral loads when using the lopinavir/ritonavir combination [100].

#### 3.2.2. Lopinavir/Ritonavir + Ribavirin

Ritonavir (RTV) and lopinavir (LPV), shown in Figure 4, are the protease inhibitors suggested in a combined form (Kaletra^®^), which is a HIV medication [20,96]. Because lopinavir inhibits the HIV protease [113], it may reduce the virus infection rate and interfere with the development of mature virus particles. Lopinavir has a short half-life and a limited bioavailability [96]. Lopinavir, an HIV protease inhibitor, can affect the maturation of viral particles and reduce the virus’s ability to spread. Conversely, Ritonavir blocks the cytochrome CYP3A4 enzyme, slows down the cytochrome P450, and reduces the lopinavir metabolism. The bioavailability of lopinavir in vivo can be increased by administering ritonavir and lopinavir together [16,96,114]. Ribavirin is a guanosine analogue that inhibits the RdRp-mediated elongation of viral RNA chains [20]. The bioavailability and in vivo antiviral activity of lopinavir are markedly increased by the combination of lopinavir and ritonavir. Compared to Remdesivir, prophylactic ritonavir/lopinavir-interferon was somewhat effective at reducing the viral load for MERS-CoV infection in mice. However, there were no significant post-infection effects on acute lung injury, viral load, or lung haemorrhage [115]. A total of 400 mg/100 mg of lopinavir/ritonavir each can be taken every 12 h for a minimum of 10 days and a maximum of 14 days. The entire dose of 400/100 mg is prescribed for children at a rate of 10 mg/kg for children weighing 15–40 kg and 12 mg/kg for children weighing 7–15 kg. Interferon-β is administered in conjunction with ritonavir/lopinavir on a 44 g for three doses every six days if the history of symptoms is less than seven days [116].

#### 3.2.3. Danoprevir

Hepatitis C protease activity is effectively inhibited by danoprevir, shown in Figure 4 [117,118]. By 2018, it had been authorized in China as an oral antiviral drug to treat hepatitis C. As a result, it was called a repurposed drug COVID-19 treatment. A triple combination of pegylated-interferon-α, ritonavir-boosted danoprevir, and ribavirin demonstrated a sustained virologic response within 12 weeks (SVR12) in Chinese patients infected with non-cirrhotic hepatitis C virus [119]. In both phase II and III clinical trials, the SVR12 rate for infected cases approached 99% when utilizing this triple combination of ribavirin, ritonavir-boosted danoprevir, and ravidasvir, an HCV NS5A inhibitor, as part of the entire oral administered therapy for non-cirrhotic HCV. Protease inhibitors are thought to offer therapeutic potential versus COVID-19 since the protease of HCV demonstrated similar function to those of SARS-CoV-2 [120].

#### 3.2.4. Darunavir

The treatment of COVID-19 in Italy has been suggested using the anti-HIV medication darunavir. In a regimen, it may be used with cytochrome P-450 inhibitors like cobicistat or ritonavir. Additionally, good anti-proliferative results against SARS-CoVs have been observed in in vitro studies [121]. In Thailand, a clinical investigation is underway to evaluate the effectiveness of darunavir in combination with other agents like hydroxychloroquine and antivirals for coronavirus patients. Additionally, a clinical trial in China explored the combination of cobicistat and darunavir. Consequently, prezcobix^®^, a fixed-dose combination of cobicistat and darunavir, could potentially be utilized in the treatment of COVID-19 patients [108]. Recently, HIV-positive individuals already on darunavir medication were exposed to COVID-19, prompting interest in the efficacy of this HIV protease inhibitor (146). However, darunavir may not be effective in preventing SARS-CoV-2 infection at the currently recommended dose of 800 mg [108].

#### 3.2.5. Atazanavir

According to an in silico investigation, atazanavir is more potent than lopinavir at the binding site in SARS-CoV-2 Mpro. Furthermore, an in vitro investigation demonstrated that atazanavir suppressed SARS-CoV-2 replication [122]. Ritonavir/atazanavir use is linked to higher lipid parameters and glucose absorption when compared to lopinavir/ritonavir use, according to research on HIV-infected individuals [123]. Studies show that atazanavir could substitute for lopinavir, as atazanavir when combined with ritonavir, will have the same effect as lopinavir alone [124].

#### 3.2.6. Saquinavir and Other Protease Inhibitors

Saquinavir, Figure 4, and other protease inhibitors, including nelfinavir, amprenavir, indinavir, and nelfinavir, serve similarly against COVID-19 as protease inhibitors due to the similarity between their chemical structures [125]. Saquinavir and indinavir were found to inhibit 3CLpro activity in SARS-CoV-2 in silico investigations. Another study discovered that indinavir, saquinavir, nelfinavir, and amprenavir suppressed SARS-CoV-2 in vitro [126]. Saquinavir, however, provides the most potent inhibition when compared to the other drugs. The drug saquinavir has been used to treat COVID-19 patients in Singapore. In a separate in silico investigation, two more possibilities, raltegravir and paritaprevir, were investigated and showed inhibitory effects on the SARS-CoV-2 3CLpro [127]. Through current screening of medicinal plant libraries, potent anti-viral phytochemicals that may function as inhibitors against SARS-CoV-2 3CLpro have been discovered [128,129]. As a result, further in vitro investigations could be used to examine the reported antiviral agents [108,127].

#### 3.2.7. Nelfinavir

Nelfinavir is an antiviral drug that uses different mechanisms to target HIV. It is an inhibitor of HIV-1 protease. The mechanism of action of nelfinavir involves binding to the HIV-1 protease active site and inhibiting the processing of functioning proteins required for HIV. According to the in vitro studies conducted during the SARS pandemic, Nelfinavir was identified as a potential agent with SARS-CoV inhibitory effects [130]. Out of the 30 examined drugs, nelfinavir provides the most potent protective effect against SARS-CoV-2. Its efficacy against SARS-CoV or SARS-CoV-2 in humans has not yet been investigated. The recommended dose for HIV is 1250 mg or 750 mg orally twice a day. However, the required dose for COVID-19 therapy is uncertain [131]. Pharmacokinetics of nelfinavir in chronic hepatic disease patients can be varied [132,133]. It causes gastrointestinal intolerance, including nausea and diarrhea [131].

### 3.3. RNA-Dependent RNA Polymerase Target, Reverse Transcriptase Inhibitors

**Figure 5:**
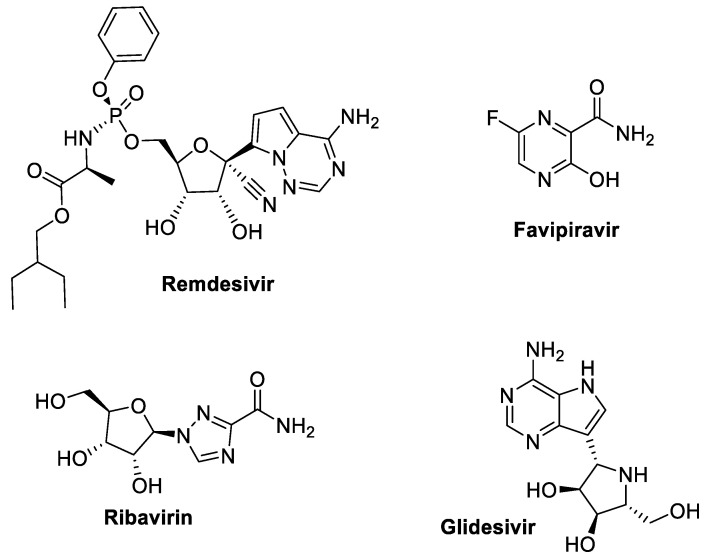
Chemical structures of RNA-dependent RNA polymerase; Remedsivir, Favipiravir, Ribavirin, and Glidesivir.

The RNA-dependent RNA polymerase (RdRp) enzyme is a vital component in the replication cycle of SARS-CoV-2, responsible for synthesizing new viral RNA strands from the viral RNA template. As an enzyme conserved among RNA viruses, RdRp is an ideal target for antiviral therapies, particularly nucleoside analogs that interfere with RNA synthesis. By inhibiting RdRp, these drugs—such as Remdesivir, Favipiravir, Ribavirin, and Glidesivir, Figure 5—effectively halt viral replication, reducing viral load and alleviating disease severity.

#### 3.3.1. Remdesivir (GS-5734, Veklury) (Gilead Sciences)

Remdesivir, Figure 5, stands as the first authorized drug specifically developed for the treatment of Ebola [134]. Ebola, being a single-strand RNA virus, faces inhibition from Remdesivir, which acts as an adenosine analogue. This inhibition targets the viral RNA-dependent RNA polymerase (RdRp), leading to either premature or delayed RNA chain termination [20,135,136,137]. Notably, Remdesivir has demonstrated antiviral activity against respiratory viral infections, including SARS-CoV-2, in in vitro settings [138]. In vivo experiments involving SARS-CoV- and MERS-CoV-infected animals revealed reduced airway inflammation and improved lung function, showcasing similar protective effects. The efficacy of post-exposure therapy is contingent on the timing of Remdesivir administration [20]. Furthermore, the licensing of nucleoside analogues for treating both DNA and RNA viruses is crucial in comprehending the mechanism of action underlying Remdesivir. However, several nucleoside analogue inhibitors have been observed to be ineffective against CoVs [139,140]. Remdesivir, a nucleoside analogue, works as an RdRp inhibitor by concentrating on the viral genome involved in viral replication. In the RdRp process, it thereby inhibits the protein complex of CoVs. The host breaks down Remdesivir to its active metabolite nortriptyline (NTP), which is then conjugated to ATP and incorporated into the developing RNA strand. Inclusion of a new strand stops the RNA synthesis and the expansion of the RNA strand once nucleotides are added. All CoVs include a proofreading process that detects and removes other nucleoside analog activity, keeping antiviral activity. Surprisingly, it has been discovered that the mutant Murine hepatitis virus (MHV) lacked proofreading capabilities and was hence highly vulnerable to Remdesivir. It is also likely that mutations that enhance proofreading or base-pairing precision will result in Remdesivir resistance. Some data suggested that Remdesivir might work via a different mechanism, permitting partial antiviral vitality to endure despite viral changes [140]. WHO has authorized/approved the emergency use of Remdesivir. WHO revised its conditional advice against Remdesivir in hospitalized patients in November 2020 and is not recommended in this situation under any circumstances [141]. Phase III clinical research on Remdesivir is crucial for obtaining the more potent antiviral drug to combat this outbreak [142]. Clinical trials in 36 of 53 patients show appropriate data in 61 hospitalized patients taking Remdesivir off-label. However, without a placebo group, these findings are difficult to comprehend. An initial randomized controlled trial was flawed, favoring Remdesivir with a non-significant trend toward shorter time to clinical changes. This trial was insufficient; however, it did show that patients treated with Remdesivir had better healing as an average recovery time of 11 days vs. placebo of 15 days. In addition, there were improvements for better survival on day 14. The research indicated disadvantages in individuals with high-flow oxygen and invasive or non-invasive ventilation, indicating that antivirals like Remdesivir might have a poor impact in late diseases where the phenotype is likely to be inflammatory. The analysis was published before the full results could be obtained via follow-up research [20]. Remdesivir is now the subject of several clinical research studies regarding COVID-19 prevention. An initial dose of 200 mg of intravenous Remdesivir is given to participants in this double-blinded, placebo-controlled study on the first day, followed by a controlled dose of 100 mg per day and up to a maximum of 10 cumulative days of treatment before release. The initial trial result is expressed as the percentage of patients in each group, employing a seven-category clinical severity scale, up to the fifteenth day following the initiation of therapy, as indicated by the United States National Library of Medicine clinical trials registry. Gilead Sciences is also supporting a Remdesivir study in patients with severe COVID-19 that will combine a primary outcome test of fever with an outcome test of oxygen normalization. In Hubei Province, China, two double-blind placebo-controlled trials included patients: one for hospitalized individuals with mild-to-moderate COVID-19 and the other for severe cases [136]. In the mild-to-moderate study, key success criteria include the normalization of body temperature, oxygen consumption, breathing rate, and cough recovery for a minimum of 72 h. Timing for health advancement is the key outcome in the extreme case study, which is presented using a six-category ratio scale from discharge to fatality [136]. It is described that Remdesivir was also found to be effective against MERS-CoV, reducing viral loads in the infected mice and restoring normal lung-based function [143]. Additionally, it is regarded as a treatment-assist agent for SARS-CoV-2 [134]. The viral load in oropharyngeal and nasopharyngeal swabs could be reduced by Remdesivir treatment for about 12 days, according to preliminary studies [144]. The combination of chloroquine, an anti-malarial drug, and Remdesivir, can successfully stop the growth of SARS-CoV-2 in Vero E6.86 cell lines in an in vitro study. The potential effect of Remdesivir in COVID-19 is being studied in clinical trials in France, the USA, and Norway. Remdesivir was used in treatment in Singapore and the USA and injected intravenously into the first patient who recovered there [145]. In a different research study, 584 participants received Remdesivir or continued receiving conventional therapy, and 533 (91%) of these patients finished the experiment. Patients in the 5-day Remdesivir group received an average of 5 days of treatment, while those in the 10-day Remdesivir group received an average of 6 days. Compared to patients getting standard therapy, patients in the 5-day Remdesivir group showed statistically noticeably higher probabilities of a better clinical status distribution on day 11 [146]. However, trial outcomes regarding safety, secondary outcomes, and viral load showed 22 of 158 Remdesivir patients died (14%), compared to ten of 78 placebo patients (13%), and there was no evidence that viral load declined differently over time in the placebo groups and Remdesivir [147]. Remdesivir’s limited oral bioavailability often limits its preventive use. Additional pharmacological measures are required to make the drug available to the outpatient population. Remdesivir inhalation Phase 1 trial and FDA approval were recently reported by the manufacturer [148]. During clinical trials, Remdesivir was administered as a freeze-dried powder injection. The dosage technique employed is as follows: on the first day, an initial dose of 200 mg of Remdesivir is supplied via intravenous dripping. Then, for the next 9 days, 100 mg is supplied intravenously as a maintenance dose [96].

#### 3.3.2. Favipiravir

Favipiravir, Figure 5, is classified as a purine analogue and was licensed for influenza treatment in Japan [149]. In vitro, it also exhibits activity against several RNA viruses, including SARS-CoV-2. Favipiravir improved the survival of the influenza A virus in mice [20]. It has been shown, in vitro, to be effective against oseltamivir-resistant A, B, and C viruses. In many RNA viruses, favipiravir is regarded as a substrate of viral RNA polymerase once it has been transformed to the active phosphoribosylated form [142]. The enzyme, in the tissue, phosphoribosylated it to its active form, favipiravir-RTP. The mechanism of action of favipiravir could be described as follows: (a) The RNA-dependent RNA-polymerase (RdRp) enzyme, which serves as a substrate molecule, misinterprets it for a purine nucleotide. As a result, its activity is inhibited, effectively ending the viral protein replication [150]. (b) By blocking further extension and integrating into the RNA viral chain, this mode of action, together with the catalytic domain’s ability to keep the RdRp enzyme active, demonstrates the wide range of activities of this molecule [151]. (c) Favipiravir is considered as a virucidal drug. During the influenza infection, it is determined to cause lethal in vitro mutagenesis. However, it is unclear whether Favipiravir has a similar action against SARS-CoV-2. Favipiravir is an orally administered drug with a mode of action similar to Remdesivir. There is less evidence to support the use of favipiravir. However, it is still emerging as a helpful drug within mild-to-moderate circumstances [152]. The advantage of favipiravir is that it is administered orally. Therefore, it can be administered as part of hospital treatment for patients with symptoms that are not critical. This drug may be utilized in several cases because mild to moderate concomitant disorders are present in many COVID-19 cases, and then care should be given at home. Favipiravir reduces viremia if given after the inception of COVID-19 symptoms. The effect of favipiravir on prophylaxis is also being studied in an ongoing study [152]. Heavy pill pressure, 18 tablets filled on the first day, and 8 tablets each day for the duration of the treatment term is its principal drawback. With the recent addition of a 400 mg dose, these concerns over the intense pill pressure are being partially allayed. A two-week prescription medication time is another drawback. Teratogenicity is the most significant side effect of favipiravir. Additionally, there were adverse outcomes such as neutropenia, elevated ALT and AST, diarrhea, and increased uric acid throughout the phase III clinical investigation in the patients in Japan. The most frequent side effects of favipiravir with COVID-19 included hepatic enzyme abnormalities, psychiatric and gastrointestinal symptoms, and blood elevations of uric acid. Although pregnant women should not take favipiravir, the side effects are often mild [96,152]. In Japan, favipiravir is authorized to treat new or recurrent influenza. It was one of the first medications for COVID-19 management to be approved [12,96,152]. The recommended favipiravir dosage for influenza in China is 1600 mg given orally every 12 h on the first day, followed by 600 mg given orally every 12 h, and on the sixth day, 600 mg given orally every 24 h [96]. Additionally, individuals with COVID-19 infections were enrolled in randomized trials with either interferon and favipiravir or baloxavir, marboxil, and favipiravir [142]. To determine whether the therapies work in concert or separately, umifenovir and other antivirals are frequently combined [142]. In an open-label, non-randomized study conducted in China, SARS-CoV-2 patients who received a double dose of interferon and favipiravir as opposed to a triple dose of interferon and ritonavir and lopinavir experienced viral clearance in 4 days vs. 11 days, and their chest X-Rays significantly improved [20].

#### 3.3.3. Ribavirin

Ribavirin (Figure 5) is known as a guanosine analogue with various RNA antiviral activities [96,153]. Many viral infections, including hepatitis C virus (HCV), respiratory syncytial virus (RSV), and other hemorrhagic viruses, are treated with Ribavirin. In vitro studies exhibited antiviral activity against SARS-CoV at 50 mg/mL concentrations. Reducing haemoglobin, which is a negative side effect that is hazardous to those who are experiencing respiratory failure [153]. Hemolytic anemia and reproductive damage are the most severe side effects of ribavirin [154]. The antiviral activities of ribavirin entail non-specific or specific chain end-up, lethal mutagenesis, and suppression of nucleotide biosynthesis [102]. For COVID-19 patients, reported by the Chinese government, ribavirin is utilized. Adults receive a dose of 500 mg administered intravenously 2 to 3 times each day for a maximum of 10 days. Ribavirin should be used with interferon or both ritonavir/lopinavir [96]. Another retrospective research study followed the adverse effects of 126 patients using 2000 mg of ribavirin as part of their treatment. Increased hemolytic haemorrhage, transaminase (40%), and bradyarrhythmia (14%) were present in certain patients [155]. Therefore, it is essential to carefully monitor the ribavirin dose when treating COVID-19 individuals [136]. During previous MERS-CoV and SARS-CoV outbreaks, ribavirin was also examined, although the results were conflicting. To stop virus replication at the micromolar stages for SARS-CoV-2, a randomized Phase II clinical trial involving 127 patients with mild to moderate COVID-19 was carried out [156]. It is more advised to combine ribavirin/interferon-β with ritonavir/lopinavir than to use just those drugs [157]. Additionally, it has been demonstrated that anti-parasitic medications such as nitazoxanide and ivermectin enhance the effects of interferon-α/β and, consequently, the immunological responses. They were investigated for COVID-19 instances without comorbidities while using ribavirin. However, the clinical trials mentioned above are insufficient to explain how ribavirin has successfully slowed the progression of the disease. On the other hand, a Phase I evaluation showed inhaled ribavirin preparation as the sole drug in the hospitalization of adult COVID-19 patients [158]. Tenofovir is a nuclear adenosine analogue used to treat HIV or HBV infections that have persisted for a long time. Tenofovir’s efficient integration can effectively stop the polymerase reaction into RNA-dependent RNA polymerase. Tenofovir is regarded as a first-line substitute for HIV prevention both before and after exposure and is thought to be a very effective component of HIV-contaminated antiretroviral therapy when combined with emtricitabine. Two-randomized Phase III clinical studies utilizing emtricitabine/tenofovir were conducted for prophylaxis prior to the exposure of healthcare workers to COVID-19, which is inconsistent with the aforesaid therapeutic approach [159].

## 4. Other Nucleoside/Nucleotide Analogs (Transcription Inhibitors)

It is possible to consider other nucleoside and nucleotide analogue medications. They either focus on treating various viral infections (such as those treated with ribavirin, sofosbuvir, tenofovir, and telbivudine) or are being professionally researched (such as galidesivir and EIDD–2801) [160]. They are anticipated to have an antiviral effect against SARS-CoV-2 because of their structural similarities to either ribavirin or Remdesivir. The FDA has granted authorization for certain drugs, including abacavir, alafenamide, tenofovir, didanosine, adefovir, ganciclovir, disoproxil, and tenofovir, as nucleoside analog reverse transcriptase inhibitors (NtRtIs). Other inhibitors include delavirdine, efavirenz, rilpivirine, nevirapine, and nucleoside reverse transcriptase inhibitors (NRTIs) such as zalcitabine, lamivudine, azvudine, stavudine, and emtricitabine can also be used to show the antiviral activity against SARS-CoV-2. More preclinical and clinical trials should be conducted to evaluate the clinical trial progress in silico trials, even though some have previously been evaluated by molecular docking [108]. As a result of interfering with the protein activity, ribavirin and sofosbuvir can be tightly bonded to the newly evolved RdRp coronavirus and eradicate the virus. It is important to note that sofosbuvir functions as a strong inhibitor of the recently discovered HCoV COVID-19 type.

## 5. Neuraminidase Inhibitors

**Figure 6:**
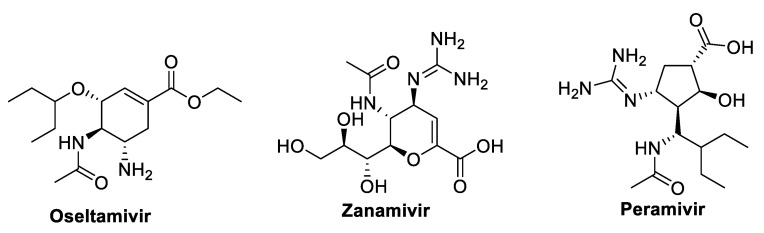
Chemical structures of neuraminidase inhibitors, such as Oseltamivir, Zanamivir, and Peramivir.

Neuraminidase inhibitors such as oseltamivir, peramivir, and zanamivir (Figure 6) are considered to be ineffective against COVID-19 and are not advised to be utilized for treatment procedures.

### 5.1. Oseltamivir

A neuraminidase inhibitor is oseltamivir (Figure 6) [113]. It is authorized for the prevention of influenza and the treatment of paediatric influenza [161]. Due to the unidentified presence of SARS-CoV-2 neuraminidase, drugs such as oseltamivir, peramivir, and zanamivir, which are neuraminidase inhibitors, are not anticipated to be effective in treating COVID-19 patients [162]. According to studies, people in Wuhan who have COVID-19 are treated with ganciclovir with oseltamivir or ritonavir/lopinavir with oseltamivir. Computational studies further supported the synergistic effects of ritonavir/lopinavir and oseltamivir in SARS-CoV-2 [163,164]. Oseltamivir was utilized in Afghanistan along with ceftriaxone and terbutaline to treat COVID-19 patients. It is revealed that three days of oseltamivir therapy significantly improved the patients’ lungs on the CT scan. In Singapore and Indonesia, oseltamivir is utilized as the COVID-19 treatment of choice [108]. Oseltamivir is administered orally for the treatment of COVID-19 and suspected patients in Chinese hospitals; however, there is currently no solid proof that it has a tangible impact on the recovery of COVID-19 patients [163].

### 5.2. Zanamivir and Peramivir

Another neuraminidase inhibitor that can be used for ventilated COVID-19 patients who are resistant to oseltamivir treatment is the zanamivir solution. Peramivir, in Figure 6, as an antiviral medication, is given intravenously. Peramivir has a certain response for patients who do not respond to zanamivir or oseltamivir [162,163]. In Chinese hospitals, oseltamivir was administered orally to patients with 2019-nCoV confirmed infections. Oseltamivir may be helpful for treating COVID-19 patients, although there is currently no concrete evidence to support this. It has recently been suggested that neuraminidase inhibitors like oseltamivir, peramivir, and zanamivir are ineffective against COVID-19 and are not advised to be utilized for treatment procedures [162].

### 5.3. M2 Ion-Channel Protein Target

#### Adamantane, Amantadine, and Rimantadine

**Figure 7:**
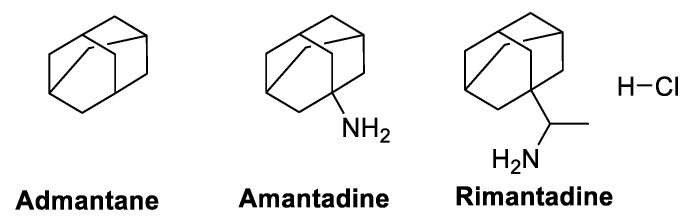
Chemical structures of M2 channel protein target such as Admantane, Amantadine, and Rimantadine.

The pH of the viral sheath must be kept constant through the M2 channel protein on the sheath. The channel, in order for steward cells to enter and pass through the trans-Golgi membrane prior to viral maturation, is essential, as well as in combating influenza viruses. A previous study demonstrated that amantadine could inhibit the HCV p7 protein, which is crucial for producing ion channels in the host cell membranes. Amantadine reportedly has a potent in vitro action against coronavirus, according to a 1973 publication [164]. A recent study demonstrated that amantadine (Figure 7) could inhibit SARS-CoV protein-membrane channel function [165]. Even though there is growing evidence that amantadine possesses antiviral potency appropriate for COVID-19 treatment, further investigation is necessary to determine its effectiveness [108,166].

## 6. Non-Antiviral Drugs Against SARS-CoV-2

**Figure 8:**
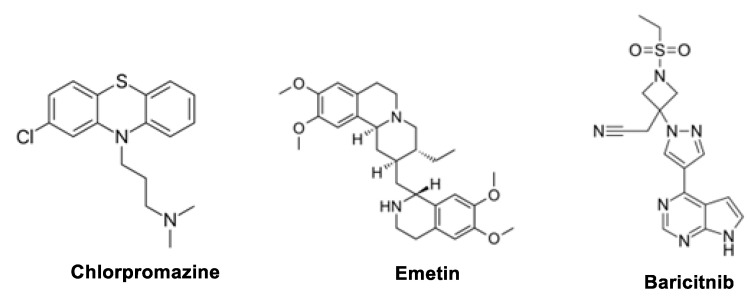
Chemical structures of some non-antiviral drugs, Chlorpromazine, Emetin, and Baricitnib.

The non-antiviral drugs such as Baricitinib, Chlorpromazine, Emetin, Figure 8, has been demostrated a wide concern against COVID-19.

### 6.1. Baricitinib

Baricitinib (Figure 8) has a high affinity against the Janus kinase (JAK) inhibitor by binding to and inhibiting adaptor-associated protein kinase 1 (AAK1). As a result, it may decrease both the inflammatory response and viral penetration brought on by SARS-CoV-2 infection. JAK inhibitors are used to treat rheumatoid arthritis and inflammatory diseases, including cancer [167]. Similar to baricitinib, JAK inhibitors such as ruxolitinib and fedratinib raise the degree of clathrin-mediated endocytosis, which may make them less efficient at lowering viral infectivity to tolerable levels. Lymphocytopenia, neutropenia, and viral reactivation are linked to its medical use [108]. Recent research has shown that the immunomodulatory drug Baricitinib and the antiviral Remdesivir have a good therapeutic effect against COVID-19 [168]. By inhibiting JAK1/JAK2, baricitinib is anticipated to alleviate the cytokine storm brought on by COVID-19. Many clinical trials have been conducted worldwide, and one of them, in which baricitinib (2–4 mg) was universally provided for 1–2 weeks, showed encouraging results. Baricitinib should only be administered with extreme caution in patients with risk factors. For its use in pregnant women, human data is insufficient [169]. The patients with renal insufficiency should therefore be cautiously examined, along with termination or dose modification [170].

### 6.2. Ivermectin; Importin α/β1-Mediated Nuclear Import Inhibitors

Ivermectin is an anti-parasitic medication approved by the FDA. It has also been demonstrated to be an effective antiviral for both the Dengue virus and HIV in humans [160,171]. A single dose of the drug can reduce the viral RNA by around 5000 times [172]. The importin α/β heterodimer pre-formed that delivers the viral protein charge nuclearly may also be isolated [108]. Ivermectin is seen as a potential therapeutic inhibitor against RNA viruses because it blocks the nuclear transport pathway of viral proteins, which is essential to the host’s antiviral response [172,173]. Ivermectin has recently been shown to have the ability to reduce viral RNA to 5000 times after 48 h of SARS-CoV-2 infection [160]. Research to establish the ideal dosage utilizing the well-established safety profile for anti-parasitic therapy is part of demonstrating the efficacy of ivermectin in COVID-19 treatment [174,175]. Further investigation is necessary to determine its efficacy against COVID-19. Additionally, ivermectin has demonstrated a broad spectrum of antiviral activity. By preventing NS3 helicase activity, it directly prevents the yellow fever virus from replicating [176]. Moreover, it stops importin α/β/1, which facilitates the passage of proteins between the nucleus and cytoplasm, which is necessary for HIV-1 replication and dengue viruses [171,176,177]. Recently, a case-controlled retrospective analysis suggested that ivermectin medication at a dose of 150 mcg/Kg might reduce the hospital admissions length and the mortality risk. Before ivermectin’s efficacy can be verified in patients with SARS-CoV-2 infection, randomized controlled clinical trials are required [108]. For the treatment or prophylaxis of COVID-19, hydroxychloroquine and ivermectin combination medicine were recommended. Because of its dual effects on viral replication and viral assembly, this combination may have a synergistic effect [178]. Although the pharmacokinetic evaluation revealed that greater dosages were required to achieve antiviral activity, administering the prescribed inhibitory concentration in humans is likewise highly difficult [179].

### 6.3. Interferon α and β

Interferons (IFNs), a broad-spectrum antiviral medication, are cytokines that activate the innate immune system in response to the viral infection. The antiviral activities and immunomodulatory of IFN can simulate protein production. IFN may also enhance the immune cells of the host cell’s particular cytotoxic action [179]. Furthermore, the interferon (IFN) response is the first crucial one of protection against viruses. Type I and type III IFN responses against viral infections are stimulated by recognition of the innate immune sensing [180]. Many disorders, such as multiple sclerosis (MS) and viral hepatitis, have already been treated with pegylated and recombinant IFN α/β. As a result, the INF proposal against COVID-19 has demonstrated widespread concern [166,181]. Therefore, it is essential to thoroughly understand the biology of coronavirus infections to include rational therapeutic strategies and assess their clinical efficacy in COVID-19 [182]. Although the cytokine development or robust chemokine or clinical investigations have revealed that the IFN reaction in patients is not mediated by significant IFN-I development [172]. When inflammatory chemokines and cytokines type I were analyzed in the serum of COVID-19 patients, no substantial amounts were discovered, but pro-inflammatory chemokines and cytokines were found to be present in high levels. By tracking the transcriptome of SARS-CoV-infected cells over time, other investigations have shown that the IFN action on the virus can be delayed rather than completely eliminated. It was also shown that IFNs can inhibit the release of pro-inflammatory cytokines [183]. A systematic evaluation of 8 types of study, involving about 116 patients, found that using interferon in combination with ribavirin induced adverse side effects, including one patient who had evident hemolysis and two patients who had an increase in pancreatic enzymes [184].

### 6.4. Teicoplanin

Teicoplanin is an antibiotic glycopeptide frequently used to treat bacterial infections. Additionally, it is being utilized in treating SARS-CoV and is on prescription drug lists for COVID-19. Teicoplanin is frequently used to treat viruses such as influenza, HIV, flavivirus, hepatitis C, Ebola, and coronavirus; SARS-CoV and MERS-CoV. It can also cure Gram-positive bacterial infections, particularly streptococcal and staphylococcal infections [185]. Teicoplanin inhibits the release of the viral genome and the viral replication cycle by cleaving the spike protein at low pH with cathepsin L, which enters the cell and targets the S protein in case of COVID-19, at the late endosomes during the early stages of viral replication [186]. The cathepsin L cleavage site has been preserved in the SARS-CoV and COVID-19 S proteins. The IC_50_ for teicoplanin in vitro is 1.66 M, which is much lower than the amount of 8.78 M for a 400 mg daily dose in human blood [162].

### 6.5. Emetine

Emetine, Figure 8, a protein synthesis inhibitor, is used to treat amebiasis as an anti-protozoan; it also works to prevent malaria by interacting with the ribosomal E site on Plasmodium falciparum. Its therapeutic utility has recently been constrained due to potential cardiotoxicity. Many RNA and DNA viruses, such as Zika virus, Cytomegalovirus, Ebola virus, Buffalo poxvirus, HIV-1, Plague of the Tiny Ruminants virus, Echovirus-1, Newcastle virus, Herpesvirus Bovine 1, Herpes Simplex Virus-2, Metapneumovirus, Rift River Fever virus, and influenza viruses, were tested for their antiviral effects [187]. Additionally, emetine was noted in vitro to suppress MHV-A59, SARS-CoV, and MERS-CoV. At 0.5 M, it was discovered to block the replication of SARS-CoV-2 effectively. In vitro, SARS-CoV-2 therapeutic plasma levels can approach 0.075 g/mL below EC50. The plasma has a toxic concentration of 0.5 g/mL. Remdesivir with 6.25 M combined with emetine with 0.195 μm may decrease the viral generation by 64.9%; more in vivo studies are warranted [160].

### 6.6. Chlorpromazine

The phenothiazine derivative chlorpromazine (CPZ), in Figure 8, which was chosen for Largactil, as the French brand name, has a wide range of properties, including antiviral, antifungal ones, anxiolytic, antiemetic, as well as immunomodulatory effects, the ability to modulate blood-brain barrier function, the ability to inhibit clathrin-mediated endocytosis, and others. It works via chlorpromazine-HCl, preventing the modulator assembly on cell surfaces and endosomes, which stops the virus from entering host cells. In addition, chlorpromazine is used to treat schizophrenia and other psychotic illnesses, tetanus symptoms, nausea, acute intermittent porphyria, chronic hiccups, and anxiety. Recently, in vitro investigations found that the CPZ had anti-MERS-CoV and anti-SARS-CoV-1 action. Without an intensive care unit (ICU), it is thought that CPZ may reduce COVID-19 infection in patients who need respiratory support [160,188].

### 6.7. Aplidin

It was declared that aplidin had antiviral action in March 2020. It is described that Multiple Myeloma is treated with aplidin on a large scale. The key to the virus’s proliferation and spread is elongation factor 1 Alpha (EF1A), which has been proven to be affected by aplidin in vitro tests. The antiviral activity of aplidin was initially investigated in human hepatoma cell lines and HCoV-229E-GFP-related viruses. The early results are generally positive for SARS-CoV-2 [189].

### 6.8. Rapamycin

Rapamycin was initially employed as an antifungal and later as an immunosuppressive medication for patients undergoing organ transplants [190]. It causes the signal transduction pathway to be interrupted downstream, which leads to the phosphorylation of mTOR. In some viral infections, such as H1N1 pneumonia, Andes virus, MERS-CoV, and HCV, mTOR inhibition prevents viral duplication and improves clinical outcomes. The well-known mechanism of action of the immunosuppressive drug rapamycin involves blocking mTOR kinase. A crucial part of viral replication is played by mTOR, and more especially by the protein complex mTORC1 that mTOR forms. It was demonstrated that rapamycin affected the PI3K/AKT/mTOR pathway, which prevented MERS infection. According to earlier research, rapamycin can regulate the production of virus particles, cytokine storms, and aid in the treatment of the illness. Consequently, it appears that rapamycin is an appealing choice for drug repurposing. Furthermore, compared to widely used antivirals, it might be a better option for COVID-19 therapy. Furthermore, the rapid mutation rate of viral RNA is unlikely to lessen its efficiency [191,192,193,194].

### 6.9. Lianhuaqingwen Capsule

In particular, lianhuaqingwen (LH) is utilized to treat influenza [195]. It reduces many symptoms such as muscle ache, headache, hyperpyrexia or fever, running nose, cough, aversion to colds, and nasal obstruction. The recommended dose is four capsules, three times a day [96]. LH was utilized by Chinese physicians to treat both mild and severe COVID-19 cases. As a potential treatment for SARS-CoV-2, LH has demonstrated effective anti-inflammatory and antiviral properties in vitro against coronaviruses [196]. Therefore, a different randomized controlled experiment (RCT) has shown that antiviral activity with LH can, in most cases, significantly relieve the COVID-19 symptoms, such as fever, fatigue, and cough. It may also significantly shorten and ease pneumonia symptoms without showing any noticeable negative effects [197]. Additionally, the antiviral potency of LH and other conventional Chinese medicines during a pandemic also effectively contributed to the treatment of SARS-CoV-2 [96].

### 6.10. Convalescent Plasma

Convalescent plasma for COVID-19, also called “survivor’s plasma”, is blood plasma obtained from COVID-19-recovered patients. Recently, the FDA approved its use in hospitalized COVID-19 patients by issuing an Emergency Use Authorization. This demonstrated that COVID-19 plasma or clean monoclonal antibodies might be extracted from patients who have totally recovered and subsequently administered to another new patient for treatment [198]. Through February 2020, about five COVID-19 patients in Shenzhen, China, received convalescent plasma treatment. Patients in this trial developed specific anti-SARS-CoV-2 antibodies from 10 to 22 days after receiving convalescent plasma therapy [199]. Reduced viral loads and improved results in sequential organ failure were observed in four of the five patients. Their virus test was still negative after the transfusion for 12 days. After two weeks of admission, the four patients were taken off artificial ventilation. However, three individuals were discharged from the hospital after over 50 days [200]. Convalescent plasma therapy has positive results, despite the trial’s extremely small sample size, and then in the US, this approach of care is advised [198].

### 6.11. Metformin

The hyperinflammatory state is a hallmark of COVID-19. Monocytes and macrophages are essential immune cells that are metabolically reprogrammed when stimulated with different stimuli, including SARS-CoV-2 spike protein [201]. Thus, drugs that regulate immunometabolism could inhibit this inflammatory response. Pre-treatment of monocytes with metformin strongly suppressed spike protein-mediated metabolic reprogramming that also suppresses inflammatory responses to SARS-CoV-2. This has potential implications for the treatment of hyper-inflammation during COVID-19 [201].

## 7. Neutralizing Antibodies for SARS-CoV-2

**Figure 9:**
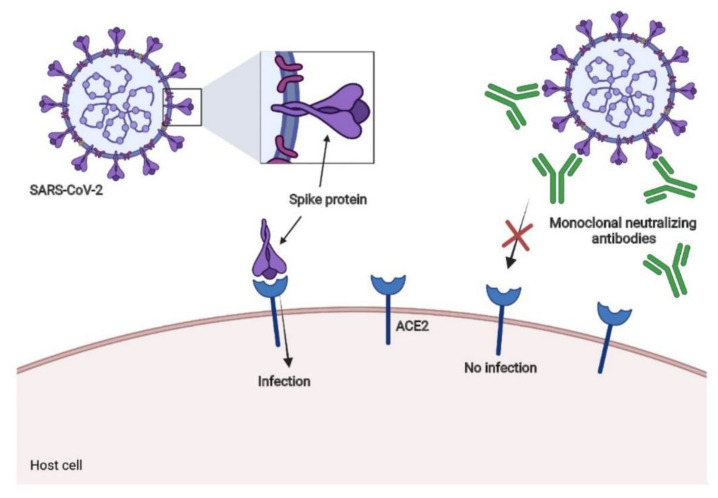
The mechanism of SARS-CoV-2-neutralizing antibodies [204].

Despite the fact that it has been effective in certain patients, convalescent plasma’s potential is still debatable. In fact, allergic reactions, transmitted infections due to transfusion (ex. HCV, HBV, HIV), and lung injury were observed in certain convalescent plasma trials. Furthermore, only a portion of plasma antibodies will be neutralizing; meanwhile the non-neutralizing antibodies will attach to non-spike protein antigens, compromising antibody responses and causing additional tissue damage. Moreover, convalescent plasma antibody titer is low, and blood resources are limited. All of these drawbacks resulted in limiting the use of convalescent plasma treatment. Conversely, anti-SARS-CoV-2 monoclonal antibodies overcome all the drawbacks of convalescent plasma by being able to specifically target the neutralizing sites and be manufactured in large quantities with ease of scalability [202]. Bamlanivimab, also known as LY-CoV555, was the first monoclonal antibody discovered to be effective against COVID-19 infection. LY-CoV555 exhibited potent binding and neutralizing action to ACE2 (Figure 9). Even at low doses, it could decrease the viral amount in respiratory tract samples [203].

Another monoclonal antibody called etesevimab has always been utilized alongside with bamlanivimab. This combination has shown more efficiency than bamlanivimab monotherapy in reducing the viral load in outpatients with mild-to-moderate symptoms in addition to reducing the risk of hospitalization and death linked to COVID-19 [205,206]. On 9 February 2021, they were approved for emergency use jointly due to their significant effectiveness in treating individuals with mild-to-moderate COVID-19. Yet, due to the Omicron variant’s high frequency, the FDA has withdrawn the use of these monoclonal antibodies for COVID-19 treatment due to its ineffectiveness compared to Omicron variance [206,207]. However, the FDA and the National Institutes of Health have approved bebtelovimab as the only effective neutralizing monoclonal antibody for the treatment of high-risk COVID-19 patients [208]. Additionally, Iketani et al. verified that, with the exception of bebtelovimab, three Omicron sub lineages demonstrated resistance to seventeen different neutralizing antibodies [209,210].

## 8. Some Recently Synthesized Compounds and Approved for COVID-19 Treatment

**Figure 10:**
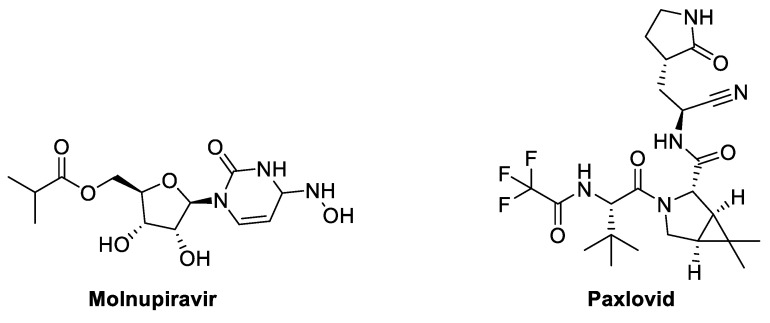
Chemical structures of Molnupiravir and Paxlovid.

The antiviral drugs such as—Molnupiravir and Paxlovid—have been demonstrated effectively against COVID-19 (Figure 10).

### 8.1. Molnupiravir (MK-4482, EIDD-2801) (Ridgeback Biotherapeutics/MSD)

Molnupiravir (Figure 10), a prodrug antiviral medicine, was used to orally treat influenza A and B viruses, was just licensed in the United Kingdom and available in November 2021. It is a synthetic nucleoside N4-hydroxycytidine (NHC) derivative that inhibits specific RNA viruses by causing copying errors during RNA replication. Compared to its precursor NHC, this medication has a higher oral bioavailability in non-human primates and ferrets. It is also effectively digested in vivo after absorption, releasing the active compound into the plasma. Using in vitro studies [211], NHC can prevent SARS-CoV-2 and other related coronaviruses [212,213]. The nucleoside analogue introduced by the viral RdRp during viral RNA synthesis leads to error catastrophe and suppression of RNA synthesis [214]. This property makes it a viable option for treating COVID-19 [215,216,217]. Molnupiravir has demonstrated effectiveness in reducing viral loads and lung pathology in Syrian hamsters and human lung-only mice, whether administered before or after SARS-CoV-2 infection [212,218]. In ferrets, post-infection treatment with molnupiravir lowered virus levels in nasal lavages and impeded transmission to untreated contact animals. Moreover, hamsters infected with the B.1.1.7 (Alpha variant) and B.1.351 (Beta variant) of COVID-19 showed resistance to SARS-CoV-2 when treated with molnupiravir. A randomized, double-blind, placebo-controlled phase 1 trial with healthy volunteers revealed that oral doses ranging from 50 to 1600 mg of molnupiravir were well-tolerated, with only a few mild side effects reported [219]. Molnupiravir is being examined in phase 3 clinical trials for COVID-19 outpatient therapy (NCT04575584), postexposure prophylaxis (NCT04939428), and inpatient therapy (NCT04575597). According to MSD release and Ridgeback Biotherapeutics [220]. Molnupiravir reduced the risk of hospitalization or death in patients with mild-to-moderate COVID-19 disease by around 50% compared to placebo and was approved by the U.K.’s Medicines and Healthcare Products Regulatory Agency [221].

### 8.2. Paxlovid (Pf-07321332)

Pfizer Inc. produced PF-07321332 (Figure 10) an oral antiviral medication. It acts as an active 3CLpro protease inhibitor. The drug combination of PF-07321332/ritonavir for the COVID-19 treatment underwent phase III research and was marketed under the name Paxlovid [131,132]. As a combination [222], ritonavir delays the cytochrome enzymes’ metabolism of the PF-07321332, keeping larger levels of the primary medication in the blood. When taken within three days of the onset of symptoms, Pfizer’s phase 2/3 results showed an 89% reduction in hospitals; it was released in November 2021 [133,223]. Ritonavir is co-administered in small doses to slow down PF-07321332 metabolism [224].

## 9. COVID-19 and Cancer

It is commonly known that viral infections increase the likelihood of developing tumors. According to estimates, viruses are the primary cause of carcinogenic illnesses, which account for 15.4% of the cases of cancer. Numerous RNA viruses have been linked to increased cancer risk, and many can result in chronic infections [225,226].

The majority of viruses use oncogenic processes that entail the continuous production of particular gene products that interact with cellular gene products to control proliferative or anti-apoptotic activity. For up to six months following a negative SARS-CoV-2 test, remnant SARS-CoV-2 nucleocapsid proteins were found in a number of extrapulmonary tissue samples, including those from the ileum, appendix, colon, lymph nodes, and liver in individuals who had recovered with COVID-19 [227,228].

Additionally, it has been noted that acute COVID-19 infection patients have changed microbiota. This change was typified by an increase in opportunistic pathogens and a decrease in commensals, or beneficial bacteria, in the gut [229].

Moreover, previous research has found that the tumor-suppressor proteins and SARS-CoV-2 spike overlap and that autoimmune cross-reactivity may be a possible mechanism behind future cancer recurrence after exposure to SARS-CoV-2.

In order to regulate p53, which poses a threat to SARS-CoV-2, it has evolved tactics similar to those of other viruses (such as the Epstein-Barr virus). Since the apoptotic signaling system depends heavily on the onco-suppressive protein p53, it has been suggested that long-term SARS-CoV-2 p53 suppression may have carcinogenic consequences [226,230].

SARS-CoV-2-caused COVID-19 infection is deemed fatal since it has a wide-ranging impact on various organs, primarily the respiratory system. It damages the neurological, cardiovascular, and pulmonary systems, among other organs, leading to organ failure. Investigations should focus on how inflammation brought on by SARS-CoV-2 affects cancer cells and the environment around tumor [231,232]. The microenvironment tumor may change due to COVID-19, encouraging cancer cell growth and reawakening the dormant cancer cell (DCC) [233,234,235]. When SARS-CoV-2 infection occurs, DCCs can reawaken and populate the pre-metastatic in the lungs and other organs, which can result in the spread of tumors. The most severe clinical effects of COVID-19 are DCC reawakening and subsequent neutrophil and monocyte/macrophage activation with an unregulated cascade of proinflammatory cytokines. The role of COVID-19 in inflammation, tumor growth, and tumor cell metastasis demand further investigation; the findings of these investigations will contribute to creating new targeted medicines for the treatment of COVID-19-positive patients as well as for the prevention of cancer [236,237,238].

## 10. Conclusions and Public Health Perspectives

The WHO declared a pandemic on 11 March 2020 in response to the new coronavirus in humans, which sparked a global threat. It is undoubtedly one of the worst public health disasters two years later. Neither the SARS-CoV-2 development nor the severe and widespread effects of COVID-19 infections were anticipated. However, the quick reaction to the COVID-19 pandemic and the responses taken by the WHO, governments, businesses, international researchers, and health authorities have strengthened public health resilience and assisted in reducing adverse effects on society. These initiatives openly disclosed data on infection rates and fatalities in terms of clinical trials. The severity of SARS-CoV-2 was lessened by open research, such as the early disclosure of the viral genome, patient trial validation of vaccine candidates, industry involvement in the development, and governments’ speedy licensing of new diagnostic tests and vaccinations. Researchers and experts are determined to develop innovative therapeutic tactics quickly and plan to combat the terrible COVID-19 epidemic. Creating new vaccines and employing some FDA-approved medications that might be tested against COVID-19 and are viewed as repurposed drugs are two therapeutic approaches. The effectiveness of many classes of currently licensed and candidate vaccines and repurposed medications (such as interferons, non-antivirals and antivirals, and anti-parasitic medicines) against COVID-19 infections has been reported. The effectiveness of several drugs has been presented and categorized according to their mechanisms of action against SARS-CoV-2. In-depth discussion was given in this study of antiviral medications, along with protease inhibitors, fusion inhibitors, M2 ion-channel protein blockers, neuraminidase inhibitors, neutralizing antibodies, and other non-antiviral drugs that may have effects against SARS-CoV-2. The recently FDA-approved drugs molnupiravir and PF-07321332 shed insight on their mode of action and eligibility as cutting-edge oral medications that fight SARS-CoV-2 by reducing hospitalizations for COVID-19 patients. Regarding the effectiveness of the vaccinations, it has been noted that Pfizer, Moderna, Fosun Pharma, BioNTech, and NIAID vaccines may be found to be the most effective to combat COVID-19. These vaccines are now being used, although they have significant drawbacks, including viral and host issues. As a result, choosing to get immunized with any of the licensed vaccinations must be carried out under medical supervision and after taking the results of current clinical studies into account. Several lessons have been learned from the pandemic, including the urgency of large-scale vaccine production and distribution, the need for point-of-care diagnostic tests and the importance of addressing the trade in wild animals and ecosystem destruction as significant contributors to the spread of infectious diseases. The COVID-19 pandemic’s lessons, such as the necessity of point-of-care diagnostic testing, the urgency of producing and distributing vaccines on a broad scale, and the significance of managing ecosystem devastation and trading in wild animals as major factors in the propagation of infectious illnesses will be crucial for addressing future dangers to the public’s health, particularly those brought on by new viruses or diseases.

## Data Availability

The datasets and materials used in this research are available upon request.
